# Status of injuries as a public health burden among children and adolescents in China: A systematic review and meta-analysis

**DOI:** 10.1097/MD.0000000000017671

**Published:** 2019-11-11

**Authors:** Ziyu Wang, Hui Chen, Taolin Yu, Siyun Liu, Ming Hu

**Affiliations:** a Department of Epidemiology and Health Statistics, XiangYa School of Public Health, Central South University, Changsha; b Department of Medicine, Betta Pharmaceuticals Co., Ltd., Hangzhou; c Department of Medical Records, Chongqing General Hospital, Chongqing, China.

**Keywords:** adolescent, children, China, injury, prevalence, systematic review

## Abstract

**Introduction:**

Injuries among children and adolescents remain a major public health problem in China. In the present study, we undertook a systematic review and meta-analysis of the published literature of epidemiological studies investigating injuries among children and adolescents in China and aimed to describe the status of injury among children and adolescents (aged 0–19 years) in China nationwide. A random-effects meta-analysis model was used to calculate the pooled prevalence of injury with 95% confidence intervals (CIs) across studies. The potential heterogeneity moderators were identified by subgroup and sensitivity analysis. A total of 775,615 children and adolescents were included in this study. Overall, the pooled injury prevalence among children and adolescents aged 0 to 19 years was 23.0% (95% CI 20.0%−27.0%) in studies using 1996 version of injury definition, and 13.0% (95% CI 10.0%−17.0%) in studies using 2004 version of injury definition. Relevant heterogeneity moderators have been identified by subgroup analysis. Sensitivity analysis yielded consistent results.

**Conclusion:**

Our study shows a moderately high injury prevalence among children and adolescents aged 0 to 19 years in China, more prevention policies and programs should be urgently developed to decrease the occurrence of child and adolescent injury.

## Abstract 2

Supplemental Digital Content is available in the text

## 1 Introduction

Nowadays, injury has become an important factor leading to death and placed a huge burden on the public health system.^[1–4]^ Worldwide, injuries accounted for more than 4.7 million deaths in 2015.^[5]^ In addition to deaths, many of those who surviving their injuries were left with temporary or permanent disabilities.^[6]^ Injuries were responsible for more than 255 million disability-adjusted life years (DALYs) in 2016, or about 10.7% of all DALYs.^[7]^ There is substantial evidence that children and adolescents are more vulnerable to injuries,^[8,9]^ and the number of deaths from unintentional injuries is staggering. Globally, every day, more than 2000 children and adolescents die from unintentional injuries,^[10]^ while road injuries ranked the first cause of death among adolescents (10–19 years).^[11]^ The impact of these injuries includes adverse effects on health and the resulting pressure on social system.^[1]^ In the United State, each year about 13,819 children and adolescents (0–19 years old) die from injuries, incurring $21.95 billion cost to the social system. And more than 0.3 million children and adolescents (0–19 years old) need hospitalization due to injuries, generating $32.14 billion cost.^[12]^

In China, the problem of injuries among young children and adolescents is also severe. Injury was the first leading cause of death of children aged 1 to 15 years, the 4th cause of death of infants under 1 years in China.^[13]^ Epidemiological research into injuries in Chinese children was always based on National Injury Surveillance System or specific surveys in localized areas.^[14,15]^ Injuries among children and adolescents have been extensively reported in China,^[16–18]^ but these surveys just focused on specific age groups or districts with limited sample size. Furthermore, published data varied considerably depending on the source(s) and population. A survey conducted in Jiangxi province is one of the largest community-based injury surveys ever conducted in China recent 5 years, including 98,335 children under 18 years of age. And the result showed 5.51% of the participants under age 18 had experienced an injury during the past 1 year.^[19]^ Up till now, making a comprehensive and systematical description of the epidemiology of injuries among children and adolescents in China is extremely difficult. However, examining the status of injuries among children and adolescents is a first step in identifying that the public health problem exists in China, efforts to improve the quality and scope of data on the burden and epidemiology of injury among children and adolescents should be undertaken in China.

To fill the knowledge gap, we undertook a systematic review and meta-analysis of the published literature of epidemiological studies investigating injuries among children and adolescents in China. We aimed to describe the status of injury among children and adolescents (aged 0–19 years) in China nationwide. Such information will enable the planning of resources and design of injury intervention strategies in China.

## 2 Materials and methods

Based on International Classification of Diseases-10, injury can be classified by fall, road traffic injury, drowning, poisoning, burns, suffocation, animal injury, electric shock, explosion injury, intentional injuries, etc. There are 2 versions of injury definition widely used in China: 1996 and 2004 versions.^[20]^ The 1996 version defined an injury that: was diagnosed as an injury by physicians and received medical treatment or was not diagnosed but the sick received emergent medical assistance from adults (teachers, parents, or others) or required the sick to rest for more than half a day because of injury. The 2004 version defined an injury that: was diagnosed as an injury by physicians and received medical treatment or required the sick to rest for more than a day because of injury. By the way, the meta-analysis was based on published articles, so the ethical approval was not necessary.

### 2.1 Literature search

We conducted the present systematic review and meta-analysis strictly following the proposed Preferred Reporting Items for Systematic Reviews and Meta-analyses Protocols statement. This systematic literature search aimed to include all studies in English or Chinese from January 2000 to December 2017 reporting on injuries among children and adolescents aged 0 to 19 years in China. Two researchers conducted searches in PubMed, OVID, EMBASE, and Chinese databases, using appropriate keywords, medical subject heading and free text terms. And search terms included “China, Chinese, child, children, preschool, infant, adolescence, adolescents, teenagers, teens, students, youth, injury, injuries, wounds” (see Text S1, Supplemental Text, which demonstrates the search strategy). Additionally, we also performed a manual search on reference lists from systematic reviews or identified articles.

### 2.2 Study selection

Eligible studies were included if they met the following criteria: cross-sectional studies with 1-year retrospect period were published between January 2000 and December 2017; study population included children and adolescents aged 0 to 19 years in China; study reported the definition of injury: 1996 or 2004 version of injury definition; and study reported the prevalence of injury and all tapes of injury. The following types of articles were excluded: study published not in English or Chinese; study repeatedly published or is a review; study lack of outcome indicators (ie, incidence rate of injury); and study with unclear or wrong date. First, the titles and abstracts of all studies identified were screened for relevance. Then a 2nd screening was based on full-text review.

### 2.3 Data extraction and quality assessment

Two independent researchers extracted data and assessed study quality. The 2 researchers agreed any discrepancies through discussion and if necessary referred the issue to a 3rd researcher. Using a study-designed standardized form, we extracted the available information from the included articles, such as first author, year of publication, geographic location, number of injured person, quality score, and stratification variables (including gender and region). Methodological quality of the included study was evaluated based on a set of appraisal guidelines that was developed by Loney et al.^[21–23]^ The tool is structured with 3 broad organizing questions and contains 8 items: sampling method, sampling frame, sample size, standard measurement, outcome assessment, response rate with refusers described, confidence intervals (CIs), and a description of subjects. Each item was assigned 1 point, and the total quality score of an article ranged from 0 to 8. A higher score indicated better the quality of the literature.

### 2.4 Statistical analysis

A random-effects meta-analysis model was used to calculate pooled prevalence of injury per year with 95% CIs across studies. However, these pooled statistics must be interpreted very conservatively since the Chi-square-based Q *P* value (*P* < .10) and *I*^2^ statistics (*I*^2^ > 50%) indicated very high heterogeneity among the studies. We considered a difference between 2 prevalence estimates to be significant if the 95% CIs did not overlap, which corresponds to a conservative estimation of statistical difference.^[24]^ A series of subgroup analyses according to age group (based on schooling age), gender, residence, sample size, and study quality score were conducted to assess the potential effect modification of these variables on outcomes. We also conducted a sensitivity analysis by removing individual studies one at a time and recalculated a pooled prevalence rate to examine whether any one study overtly influenced the pooled effect size. Finally, potential publication bias was identified by Egger test. All statistical analyses were performed using STATA software version 13.0 and Review Manager version 5.3. Unless otherwise specified, *P* < .05 was defined as statistically significant for all tests.

## 3 Results

### 3.1 Study characteristics

**Figure 1:**
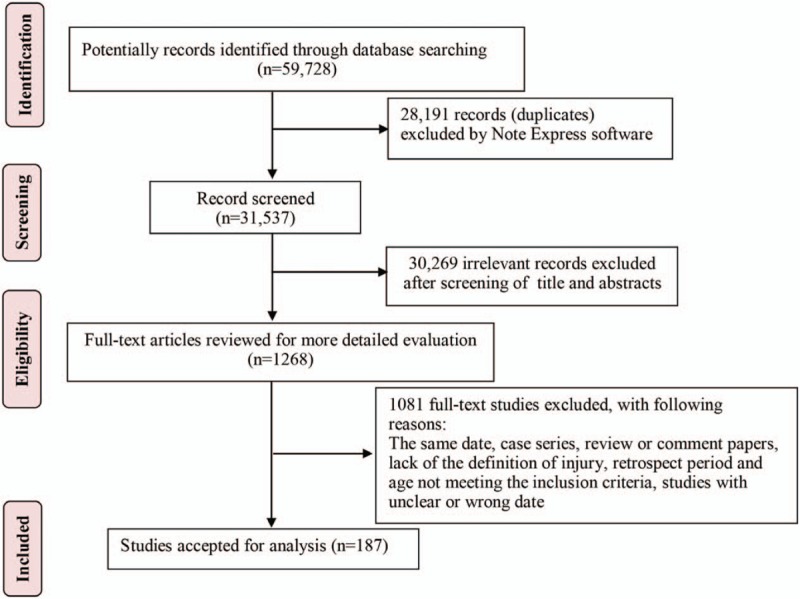
Preferred Reporting Items for Systematic Reviews and Meta-analyses Protocols (PRISMA) statement flow diagram.

We identified 59,728 papers through our systematic literature search, after removing duplicates and irrelevant records, 1268 papers were left to be assessed. A total of 1081 papers were excluded for not meeting the inclusion criteria, leaving 187 papers including 775,615 objects for final analysis (Fig. 1). Of these 187 studies (see Text S2, Supplemental Text, which includes these 187 references), 145 studies used 1996 version of injury definition while 42 studies used 2004 version of injury definition. The quality score ranged from 4 to 8 points. Among them, 27 studies scored 4 points, 47 studies scored 5 points, 80 studies scored 6 points, 29 studies scored 7 points, and 4 studies scored 8 points (see Table S1, Supplemental Content, which summaries the characteristics of included studies).

### 3.2 Pooled injury prevalence among children and adolescents

Significant difference was found from meta-analysis for the pooled injury prevalence between 2 versions of injury definition. In studies using 1996 version of injury definition, the injury prevalence among children and adolescents aged 0 to 19 years reported by individual studies ranged from 1.0% to 80.4%. Based on the 145 included studies, the pooled injury prevalence was 23.0% (95% CI 20.0%–27.0%). However, significant heterogeneity was found (*I*^2^ = 100%). With regard to specific injury types, the present meta-analysis showed the first 3 types of injury were fall, collision/crush, and sharp instrument, the prevalence were 9.0% (95% CI 8.0%–11.0%), 5.0% (95% CI 4.0–6.0%), and 4.0% (95% CI 3.0%–4.0%), respectively. In studies using 2004 version of injury definition, the injury prevalence among children and adolescents aged 0 to 19 years reported by individual studies ranged from 1.0% to 80.4%. Based on the 145 included studies, the pooled injury prevalence was 13.0% (95% CI 10.0%–17.0%), with substantial heterogeneity (*I*^2^ = 100%). Furthermore, the first 3 types of injury were fall, collision/crush, and sharp instrument injury, the prevalence were 6.0% (95% CI 4.0%–8.0%), 3.0% (95% CI 1.0%–7.0%), and 1.0% (95% CI 1.0%–2.0%), respectively.

### 3.3 Subgroup analysis

**Figure 2:**
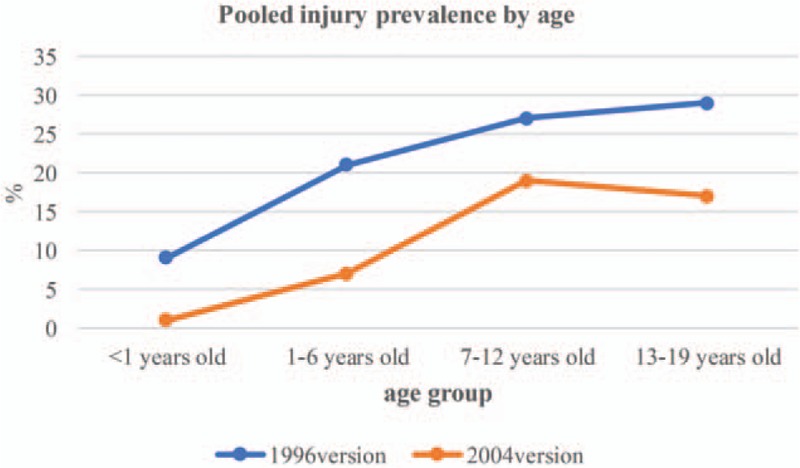
Pooled injury prevalence by age groups.

Subgroup analyses for pooled injury prevalence among children and adolescents aged 0 to 19 years are summarized in Table 1. In studies using 1996 version of injury definition for the pooled injury prevalence, after subgroup analysis, age group (test for subgroup difference [TSD]: *I*^2^ = 70.6%) and gender (TSD: *I*^2^ = 84.8%) were identified as the relevant heterogeneity moderators. There was an increasing trend in injury prevalence among children and adolescents along with the age (Fig. 2). Furthermore, boys suffered more injuries compared with girls, the pooled estimates were 28.0% (24.0%–33.0%) and 21.0% (18.0%–25.0%) for boys and girls. In studies using 2004 version of injury definition for the pooled injury prevalence, after subgroup analysis, age group (TSD: *I*^2^ = 97.3%), residence (*I*^2^ = 71.1%), and study quality score (TSD: *I*^2^ = 77.4%) were identified as the relevant heterogeneity moderators. The pooled injury prevalence was highest among children and adolescents aged 7 to 12 years (19.0% [12.0%–31.0%]). Compared with children and adolescents in urban areas, those who were in rural areas had more higher injury prevalence, the pooled estimates were 17.0% (12.0%–23.0%) and 10.0% (6.0%–15.0%) for rural areas and urban areas, respectively. Finally, the pooled estimate in studies with quality score <5 points (29.0% [14.0%–63.0%]) was higher than studies with quality score ≥5 points (12.0% [9.0%–16.0%]).

**Table 1:**
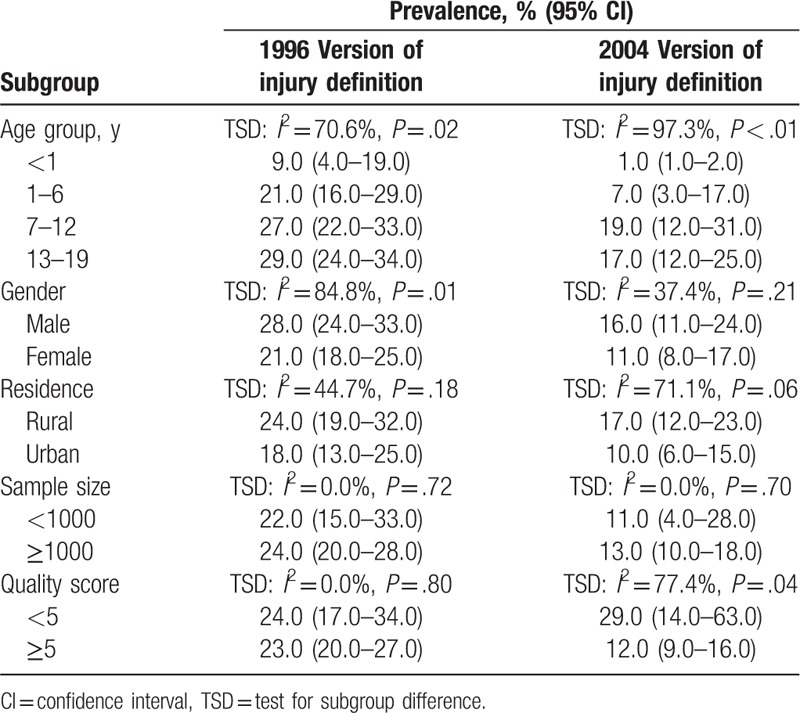
Subgroup analysis of the pooled injury prevalence among children and adolescents aged 0 to 19 years.

### 3.4 Publication bias and sensitivity analysis

Sensitivity analyses were performed for above meta-analysis. By serially excluding each study from the analyses, the pooled estimates varied slightly, indicating that the result was relatively stable. However, Egger tests showed that publication bias existed.

## 4 Discussion

This systematic review and meta-analysis synthesized the prevalence of injuries among children and adolescents (aged 0–19 years) in China as reported by 187 different studies including 775,615 objects during the period of 2000 to 2017. The pooled injury prevalence was 23.0% in studies using 1996 version of injury definition, 13.0% in studies using 2004 version of injury definition.

According to previous studies, injury prevalence among school children aged 6 to 11 years was estimated to be 17.3% in Europe, and 24.75% in Bulgaria,19.0% in Lithuania, 18.8% in Turkey, 15.7% in Romania, 13.1% in Netherlands, 30.4% in West Germany, and 22.3% in East Germany.^[25]^ And high annual injury prevalence was found among adolescents in 4 Southeast Asian countries, 42.2% adolescents were reported 1 or more serious injuries within the past 12 months for all countries, 27.0% in Myanmar, 37.2% in Sri Lanka, 45.9% in Indonesia, and 46.8% in Thailand.^[26]^ Above all, we can observe the injury prevalence among children and adolescents in China is lower than it in Southeast Asian countries, but higher than it in Europe.

Several factors could explain the variability of injury rates reported in studies from different countries, such as data collection methods, cultural, and lifestyle characteristics.^[27]^ But the most important factor may be differences in the economic level of different countries. High injury burden often occurs in low-income and middle-income countries due to the weak safety infrastructure, few regulatory, and societal response to injuries.^[28–31]^ In China, to reduce the death and disability caused by pediatric injuries, “*the 2016–2020 child injury prevention project*” led by Chinese Center For Disease Control And Prevention was launched in Beijing recently.

Also some differences arouse from the definition of injury. In our study, injury prevalence varied by different versions of injury definition. In 2004 version of injury definition, “the children received emergent medical assistance from adults (teachers, parents, or others)” was removed, for which, those children and adolescents with slight injuries may be excluded. It is the major reason why the injury prevalence decreased as using 2004 versions of injury definition. Meanwhile, that “rest for more than a day before returning to normal activity” replaced “rest for more than half a day before returning to normal activity” also lead to underestimating the rate. The definition “injuries that were serious enough to require medical attention by a doctor, nurse, or dentist” was also used widely in many countries.^[25,32]^ The criteria is simple and operational, but it also may underrate the incidence of injury, especially in poor areas lack of medical resources. Globally, injuries require an accepted and general definition for better surveillance and prevention. It remains to be further improved in future studies.

When subset by gender and age, we observed a higher risk of injury among boys compared with girls. Sex differences in injury incidence have been reported in many countries and the prevalence of injury was usually found to be higher in boys than in girls.^[27,33–36]^ Compared with girls, boys are more likely to be exposed to outside dangers that elevate risk of injury. Besides, girls may receive more active supervision from their guardians than boys. So, more attention should be paid to boys in injury prevention. The data from our review also indicated an increasing trend in injury incidence among children and adolescents along with the age.

Regarding the type of injury, we found fall as the most common type of injury. Fall was also the leading cause of injuries among young people in most of the community-based studies conducted in many countries.^[37,38]^ Each year, falls occurring in children are responsible for over 6.8 million DALYs lost worldwide.^[39]^ However, falls can be preventable, evidence from Canada showed the implementation of effective prevention strategies can reduce 20% falls among children under 10 years of age.^[39]^ Given to the severe outcome of falls, it is necessary to target protecting our children and adolescents from fall-related injuries as top priorities.

In our study, the injury prevalence among children and adolescents was higher in rural areas than in urban areas. Poverty is an important socioeconomic factor leading to child injury,^[28]^ children and adolescents from poor regions and families are more vulnerable to injuries. A previous study conducted in China found the evidence of wealth disparity for animal bites, falls, and road traffic injuries.^[19]^ For example, in rural areas of China, households often have guard dogs, children who live in rural areas may be more likely to be exposed to their own or neighbors’ or street dogs and therefor easily experience animal bite injuries. The finding indicated special attention should be paid to injury control and prevention on rural children and adolescents.

Our study has several strengths. As far as we know, our study was the first meta-analysis included a large sample size to estimate the pooled injury prevalence among children and adolescents, giving it sufficient statistical power. Moreover, we calculated the pooled prevalence injury in 2 versions of injury definition, improving the comparability between different genders, age groups, and regions.

However, we have to acknowledge that there are certain limitations of our study. First, all the included studies were cross-sectional and injuries were self-reported or parent-reported, which may have introduced recall bias and reporting bias. Second, overall estimates must be interpreted with caution because of substantial heterogeneity. Nevertheless, we were able to use subgroup analysis to explore the major source of heterogeneity. Our subgroup analyses have identified main heterogeneity moderators, including age group, gender, residence, and study quality score. In addition, after sensitivity analysis that removed 1 study at a time and calculated the pooled prevalence for the remaining studies, the result with slight changes was stable and reliable. Despite the above limitations, our findings have implications for practice, policy, and research. Third, in our review, more than half of the included studies were conducted in eastern regions of China. Moreover, little data was available in China's poorest remote areas, such as Tibet, Xinjiang, etc. So, our results may not be representative in poor and remote areas of China.

## 5 Conclusions

In conclusion, our study shows a moderately high prevalence of injuries among children and adolescents aged 0 to 19 years in China. This represents a serious public health issue in China, more prevention policies and programs should be urgently developed to decrease the occurrence of child and adolescent injury.

## Acknowledgments

The authors thank the World Health Organization (Geneva) and the Centers for Disease Control and Prevention (Atlanta) for making the data available for analysis. And the authors also thank the editors and reviewers for their suggestions.

## Author contributions

**Conceptualization:** Ziyu Wang, Hui Chen, Taolin Yu, Siyun Liu, Ming Hu.

**Data curation:** Ziyu Wang, Taolin Yu, Siyun Liu, Ming Hu.

**Formal analysis:** Ziyu Wang, Taolin Yu, Ming Hu.

**Funding acquisition:** Ming Hu.

**Methodology:** Ziyu Wang, Hui Chen.

**Software:** Ziyu Wang.

**Supervision:** Taolin Yu.

**Visualization:** Ziyu Wang.

**Writing – original draft:** Ziyu Wang.

**Writing – review & editing:** Ziyu Wang.

## Supplementary Material

Supplemental Digital Content
