# Supplementary material for: Status of injuries as a public health burden among children and adolescents in China: A systematic review and meta-analysis
Source: Medicine (Baltimore). 2019 Nov 11;98(45):e17671. doi: 10.1097/MD.0000000000017671 (PMC6855559; doi:10.1097/MD.0000000000017671)
Supplement: Supplemental Digital Content [file medi-98-e17671-s001.doc]

**Text S1. Search strategy**

Chinese databases:

CNKI: injury[Title] (2000-01-01 to 2017-12-31) (N=14019)

Wan Fang data: injury[Title] Date:2000-2017 (N=23259)

VIP: injury[Title] Date:2000-2017 (N=16185)

Sino Med: injury[Title] Date:2000-2017 (N=4597)

PubMed:

#1 (China[Title/Abstract]) OR Chinese[Title/Abstract]

#2 China[MeSH Terms]

#3 #1 OR #2

#4 (injury[Title]) OR injuries[Title]

#5 (Wounds and Injuries[MeSH Terms])

#6 (((Wounds and Injuries[MeSH Terms]))) OR ((injury[Title]) OR injuries[Title])

#7 ((((((((((child[Title/Abstract]) OR children[Title/Abstract]) OR preschool[Title/Abstract]) OR infant[Title/Abstract]) OR Adolescent[Title/Abstract]) OR Adolescence[Title/Abstract]) OR Adolescents[Title/Abstract]) OR Teenagers[Title/Abstract]) OR Teens[Title/Abstract]) OR Students[Title/Abstract]) OR Youth[Title/Abstract]

#8 (((child[MeSH Terms]) OR infant[MeSH Terms]) OR Students[MeSH Terms]) OR Adolescent[MeSH Terms]

#9 #7 OR #8

#10 ("2000/1/1"[Date - Publication] : "2017/12/31"[Date - Publication])

#11 #3 AND #6 AND #9 AND #10

(N=1224)

OVID:

#1 (China or Chinese).ti. or (China or Chinese).ab.

#2 (injury or injuries).ti.

#3 (child or children or students or preschool or infant or Teenagers or Adolescents or Adolescence or Adolescent or teens or youth).ti. or (child or children or students or preschool or infant or Teenagers or Adolescents or Adolescence or Adolescent or teens or youth).ab.

#4 #1 AND #2 AND #3

#5 limit 4 to yr="2000 - 2017"

(N=234)

Embase:

#1 China: ti OR Chinese: ti OR China: ab OR Chinese: ab

#2 injury: ti OR injuries: ti

#3 child: ti OR children: ti OR students: ti OR preschool: ti OR infant: ti OR teenagers: ti OR adolescents: ti OR adolescence: ti OR adolescent: ti OR teens: ti OR youth: ti OR child: ab OR children: ab OR students: ab OR preschool: ab OR infant: ab OR teenagers: ab OR adolescents: ab OR adolescence: ab OR adolescent: ab OR teens: ab OR youth: ab

#4 #1 AND #2 AND #3

#5 #1 AND #2 AND #3 AND [2000-2017]/py

(N=210)

**Text S1.References for studies included in the meta-analysis**

1. Wang JX, Han YX, Liu QY, et al. An epidemiological study on injury among students in middle schools affiliated to factories in HanDan City. *Modern Preventive Medicine*, 2000; *27(2)*, 198-199.

2. Hu LR, Chen GJ, Liu JL, et al. A study in incidences and determinants of injuries among teenagers. *Modern Preventive Medicine*, 2000; *27(2)*, 154-157.

3. Han YX, Gao YM, Wang JX, et al. Epidemiological survey of unintentional injury in school children in Handan City. *J Per Med Inf*, 2000 *(1)* :10-12.

4. Wang ShY, Guo Ch, Zhang GY, et al. A study on incidence of injury and its socio-economic loss in children and young adults. *Chin J Prev Med*, 2000, *34(4)*:203-205.

5. Wu KM, Xu YH, Li L, et al. Incidence of injury and its risk factors in preschool children in the urban areas of Chengdu. *Chin J Prev Med*, 2001 , *35 (3)* :181-184.

6. Liu L, Liu XX, Wen QSh, et al. Research on unintentional injuries and its affecting factors in preschool children at urban area of Guiyang City. *CJCHC*, 2001, *9(2)* :91-93.

7. Yang BH, Li J, Xiao HP, et al. An epidemiological survey on injuries among students in primary and middle school of ChenZhou City. *Modern Preventive Medicine*, 2001, *28(2)*:152-154.

8. Ye DQ, Huang F, Chen WH, et al. An investigation on injuries among middle and primary school students in Huaiyuan County. *Chin J School Health* , 2001*(2)*:131-132.

9. Li YZh, Incidence of injury in children aged 0~14 at rural area of Tongjiang County. *Chinese Rural Health Service Administration*, 2002, *22(8)*:55-57.

10. Xu YQ, Luo Q, The related factors study of unintentional injuries in rural children under six in Shiyan City. *Journal of Hubei University of Medicine*, 2002,*(5)*:284-285.

11. Ni Y, Yang HH, Li XX, et al. A prevalence study on infant injury in Shantou. *Chinese Journal of Disease Control & Prevention*, 2002, *6(1)*:77-78.

12. Huang WQ, An epidemiological survey and burden analysis of injury on adolescent students. *Chin J Public Health*, 2002, *18(6)*:751-752.

13. Yao YSh, Epidemiology study on distributive characteristics of injuries in middle school students of Wannan Area. Diss, *Medical University Of Anhui*, 2002.

14. Liang HY, Zhang QX, Zhang ShY, et al. Investigation on characteristics of injuries epidemiology of students from middle and primary schools in Shenzhen. *Chin J Public Health*, 2002, *18(10)*:1170-1171.

15. Hu ShF, Li ZhY, Xi GP, et al. Incidence of injury among primary and middle school students in Pingliang Area. *Chin J School Health*, 2002 , *23 (6)* :555-556.

16. Zhao JH, Huang Ch, Liu MZh, et al. Risk factors for accidental injuries among middle and primary schools students in Ningxia. *Chin J School Health*, 2002 , *23 (6)* :504-505.

17. Li AnL, Zheng L, Ma Y, et al. Epidemiology study on characteristics of injuries in primary school students. *Chin J Epidemiol*, 2003 , *18 (8)* :308-309.

18. Liu XL, Xu Q, Cheng XP, Investigation on unintentional injuries in primary school students in Jinzhou area. *Chin J Public Health*,  2003 , *19 (1)* :76-77.

19. Fu M, A study on incidence of unintentional injuries and its affecting factors in kindergarten children in Gongbei community of Zhuhai city. Diss. *Jinan University*, 2003.

20. Zhao JB, Feng WJ, Wu ShL, et al. A study on injuries and its affecting factors among primary school students in Harbin. *Chin J of PHM*, 2003 , *19 (3)*: 255-257.

21. Liu WG, Zheng H, Lei J, et al. An epidemiological survey on injuries among students in primary school of Yinchuan City. *Journal of Medical Pest Control*, 2003 ,*19 (1)* :11-13.

22. Zhao JB, Feng WJ, Wu ShL, et al. A study on injuries and its affecting factors among middle school students in Harbin. *Chin J public health*, 2003 , *19 (8)* :972-972.

23. Wang YZh, Zhou MR. A study on injuries and its affecting factors among students in two technical secondary schools of Qinghai province. *Chin J School Health*, 2003 , *24 (2)* :147-148.

24. Shi MC, Zhang LSh, Xu XY, et al. An epidemiological survey on injuries among students aged 2~12 in the fifth division of agriculture of Xinjiang production and construction group. *Chin J public health*, 2003 , *19 (1)* :71-72.

25. Li LP, Wang S, Huang G, et al. A survey on injury incidence in school children in Shantou city, China. *Biomed Environ Sci*. 2003, *16(2)*:180-186.

26. Wang J, Wang XQ. A study on incidence of injury among primary and middle school students in Jiuquan prefecture. *Chin J School Health*, 2003 ,*24 (5)* :543-544.

27. Liu AiM. An investigation on injuries among middle and primary school students in Dafeng city. *Chin J School Health* , 2003 , *24 (6)* :658-659.

28. Xing GY, Wang JX, Wang J, et al. An investigation on injuries among middle and primary school students in Luozhuang District, Linyi. *Lit & Inf Prev Med*, *2003(05)*:524-525.

29. Huang F, Hao JW, Ye DQ, et al. Investigation on injuries among middle and primary school students of countryside in Suzhou. *Chin J School Doctor*, 2003 , *17 (5)* :398-400.

30. Zhong JM, Yu M, Cong LM, et al. A cross-sectional study on injuries in primary and secondary school students in Zhejiang province. *Chin J School Health*, 2003, *24 (2)* :120-122.

31. Ye Y. Research on socio-psychological factors for injures in junior middle school students. Diss. *Huazhong University of Science and Technology*, 2004.

32. Sun Y, Zhang ML. Epidemiology study on characteristics of injuries in primary school students in Linyi city. *Chin J School Doctor*, 2004 , *18 (5)* :420-425.

33. Pang ShL, Xue L, Guan WJ, et al. Epidemiological survey on injuries among middle school students in Tangshan. *Chin J Nat Med*, 2004, *6(1)*:19-21.

34. Wu XH, Chen JH, Yang ChC, et al. An epidemiological investigation on injury in pupils in Huangshi city. *Chin J Dis Control Prev*, 2004, *8(6)*:591-592.

35. Shi Y, Jiao ShF, Xie J, et al. An epidemiological survey on the status and risk factors of unintentional injury among children in the urban and rural area of Beijing. *Chin J Dis Control Prev*, 2004 , *8 (6)* :588-590.

36. Lin Y, Wang H, Jiang XM, et al. Analysis of 0~6 year-old child injury in Shenzhen community. *Chinese Primary Health Care*, 2004 , *18 (12)* :53-54.

37. Dai LP, Wang QSh, Zeng MF. An epidemiological study of injuries among middle school students in Loudi city. *Chin J School Health*, 2004 , *25 (3)* :297-299.

38. Zhang Y, Sun YH, Yu DX, et al. Negative binomial regression analysis of relative factors of unintentional injury among middle school students. *Chin J Dis Control Prev*, 2004 , *8 (6)* :528-530.

39. Xiao L. Analysis of unintentional injury among middle and primary school students in Nanjing. *Chin J School Health*, 2004 , *25 (3)* :366-367.

40. Li ZhH. A Cross-sectional study on injury in residents from the countryside of HuangHe Delta rural areas. Diss. *Shandong University*, 2004.

41. Chang JX, Cong PF, Peng N. Investigation on injuries among middle and primary school students in Rongcheng, 2003. *Prev Med Trib*, 2004 , *10 (6)* :665-665.

42. Zhang Y. Evaluation of injury prevention programme based on health education for elementary school children. Diss. *Anhui medical university*, 2004.

**43.** Xu ShD. The study on the relationship of incidence of unintentional injuries and behavioral status in children at 3~7. *Wuhan University*, 2005.

44. Zhang D, Bao ChH, Feng ChR. Investigation for accidental injury of primary and middle school students in Xicheng district of Beijing city. *Modern Preventive Medicine*, 2005 ,*32 (11)* :1470-1472.

45. Wang H, Liu YX, Liu Y, et al. Analysis of 0~14 year-old child injury in Henggang town of Shenzhen city. *Chinese Primary Health Care*, 2005, *19 (6)*: 24-25.

46. He ZhH, Cheng ZhX. An epidemiological survey on children’s injuries among children aged 0~14 in Tianhe district of Guangzhou. *Chin J School Health*, 2005 , *26 (2)* :116-118.

47. Li SF, Liu RT, Zeng XQ. A case-control study of the affecting factors for injuries among children aged 0~14 in Fangcun district. *Maternal and Child Health Care of China*, 2005 ,*20 (5)* :622-624.

48. Wei Y, Yang BF, Tang ZhY , et al. Investigation on injuries among middle and primary school students of 11 towns in Rencheng district. *Chin J of Behavioral Med Sci*, 2005 , *14 (9)* :813-813.

49. Zhang YF, Zhu CQ, Zhu B, et al. Analysis of under 15 year-old child injury in Shaoxing city. *Zhejiang Prev Med*, 2005 , *17 (2)* :31-32.

50. Chu JF, Zhu MSh. Present situation of injury among middle and primary school students in Xiaoshan district, Hangzhou city. *Chinese Journal of Health Education*, 2005 , *21 (12)* :942-944.

51. Liu L, Shang YX, Liu XJ. An epidemiological survey on unintentional injuries among students in primary and middle school of Yinchuan. *Journal of Ningxia Medical University*, 2005 , *27 (1)* :14-16.

52. Xu GZh, Wang RY, Ye Zh, et al. Study on characteristics of injuries epidemiology of students from middle and primary schools in urban and rural areas of Ningbo city. *Chin Prev Med*, 2005 ,*6 (6)* :521-523.

53. Chen X, Liu XN, Shi YL, et al. Epidemiological survey of unintentional injury among children aged 0~14 in Xuzhou. *Chin J School Doctor*, 2005 , *19 (1)* :33-34.

54. Yan XY. Unintentional injuries and affecting factors among pre-school children and in Yueyang city and the knowledge, attitude and practice of childhood injury among the parents. *Central south university*,2006.

55. Xiong JF, Yang L, Zhou HB, et al. Analysis of features of injury in primary school pupils and middle school students and influential factors in Longgang District, Shenzhen City. *China Tropical Medicine*, 2006 ,*6 (9)* :1693-1694.

56. Xu HQ, Zhang D. An epidemiological investigation on injury among middle and primary school students in rural areas of Conghua. *South China J Prev Med*, 2006 ,*32 (6)* :53-55.

57. Xu W, Yang WP, Wu YSh, et al. Study on epidemic characteristics and disease burden of injuries among the population of Zhabei District. *J Environ Occup Med*, 2006 ,*23 (5)* :381-384.

58. Ma DH. Incidence of injury among middle and primary school students in Yanbian County. 　*Chin J Sch Health*, 2006 , *27 (12)* :1090-1091.

59. Shao YQ, Zhang YY. Epidemiology study on characteristics of injuries among students in a school for the children of migrant workers. *Chin J Sch Health*, 2006 , *27 (9)* :814-814.

60. Zhang Y, Li HF. An epidemiological investigation on unintentional injury in teenagers. *Journal of Community Medicine*, 2006 , 4 (12s) :11-13.

61. Yu Y, Sun GCh, Xu G, et al. Study on epidemic characteristics and risk factors of injuries among middle and primary school students in a district of Nanjing. *Chin J School Doctor*, 2006 , *20 (5)* :489-491.

62. Zhou DD, Lu W, Li YH, et al. The injury pattern in children aged 0-14 years in Shanghai. *J Environ Occup Med*, 2006 , *23 (3)* :234-237.

63. Chu QP, Ge XW, Kong QL, et al. A Cross-sectional study on unintentional injury among students in middle school of Xuzhou. *Chin J Sch Health*, 2006 ,*27 (12)* :1092-1093.

64. Tan ShK, Wu QJ, Ma YJ, et al. An epidemiological survey on unintentional injuries among Han and Zhuang students in Bbaise area. *Journal of Guangxi Medical University*, 2006 , 23 (1) :151-153.

65. Nie ShP, Li HK, Xu YJ, et al. Analysis on the risk behaviors related to injury of adolescent in Guangdong city. *Chin J Sch Health*, 2006 ,*27 (11)* :930-931.

66. Zhao D, Li LP, Lin WJ, et al. A cross-sectional study on injuries among island residents. *Chin J Dis Control Prev*, 2007 ,*11 (5)* :471-474.

67. Xu WM, Liu GJ, Zhou JH, et al. The investigation on epidemic characteristic and effect factor of injury in primary school students in Hangzhou, *Chinese Primary Health Care*, 2007 ,*21 (3)* :66-67.

68. Li LJ. Analysis of the investigation of the accident injuries in children aged between 0 - 10 years old in Wuhan city. *J Clin Pediatr*, 2007, *25 (3)* :213-215.

69. Ding YP. Investigation on injuries among middle and primary school students in Huairou district of Beijing. *Occup Health*, 2007 , *23 (5)* :375-377.

70. Yang LX. A Study on epidemiological characteristics and intervention of unintentional injuries in primary and middle school students of Mentougou District. Diss, *Capital Medical University*, 2007.

71. Cai LG, Cui YQ, Song YP, et al. Status of knowledge and behavior about accidental injury among pupils in Changping District of Beijing. *Chinese Journal of Health Education*, 2007, *23 (5)* :384-385.

72. Wang RSh, Guo JF, Liu H. Investigation on unintentional injuries among primary school students. *Modern Preventive Medicine*, 2007 ,*34 (1)* :85-85.

73. Xue L, Pang ShL, Liu RG, et al. Investigation on intentional injuries of pupils in rural areas of Tangshan city. *J Pub Health and Prev Med*, 2007,*18 (6)* :45-47.

74. Xiang B. Survey on accidental injuries among 1 690 children in Wuhan. *Chin J Dis Control Prev*, 2007 ,*11 (3)* :314-315.

75. Zhang YY, Wang FY. Investigation of injury about pupils in Hezuo City of Gansu Province. *Chinese Journal of Health Education*, 2007 , *23 (1)* :28-29.

76. Xing YP, Jiu ShW, Shi HF. Epidemiological study on injury among middle school students in Shangqiu. *Chinese Journal of Health Laboratory Technology*, 2007 , *17 (6)* :1082-1083.

77. Yao YSh, Jin YL, Ye DQ. A cross-sectional investigation on unintentional injury in preschool children from the southern parts of Anhui Province. *Chin J Epidemiol*, 2007 , *28 (12)* :1240-1241.

78. Wang YL, Xia Q, Huang HM, et al. Epidemiological study on injuries among primary school students in Yangpu District of Shanghai. *Chin J Sch Health*, 2007 , *28 (3)* :282-282.

79. Li N, Wang P, Liu MJ, et al. Cross-sectional study on injuries among children and adolescents in Liaoning Province. *Chin J Dis Control Prev*,  2007 , *11 (3)* :274-276.

80. Yang YM, Chen LSh, Yang J, et al. A cross-sectional study on injury in minors from Sunan area of Jiangsu Province. *Modern Preventive Medicine*, 2007 , *34 (11)* :2096-2097.

81. Zhu LP. Epidemiological Survey of Child Injury in Jiangxi Province. *Chin Prev Med*, 2007 ,*8 (5)* :521-526.

82. Li ShSh, Zhang ChG, Cui HW, et al. Analysis on epidemic characteristics of injuries among the population of Kangjian community in 2006. *Chin J Dis Control Prev*, 2008 ,*12 (5)* :508-510.

83. Li L. Study of unintentional injury children’s behavior characteristics of preschool children in urban of Jinan. Diss, *Shandong university*, 2008.

84. Wu DN, Li LT, Wu HSh, et al. Study on the injury and health risk behavior among 1011 sensor high school students of grade one and two in Shenzhen. *Modern Preventive Medicine*, 2008 ,*35 (20)* :3982-3984.

85. Zhang XP, Wang X. A study on incidence of accidental injuries and its affecting factors among middle school students in Jiujiang. *Chin J Public Health*, 2008 ,*24 (11)* :1319-1320.

86. Zeng ChJ, Luo Q. Retrospective investigation on factors influencing accidental injury to city students. *Chinese Journal of Healthy Birth & Child Care*,  2008 , 14 (1).

87. Xu ZN, Zhu XZh, Gong ShB. Investigation on risk behaviors of injuries in adolescents in Qidong city. *Chin J Sch Health*, 2008 , *29 (12)* :1089-1091.

88. Cheng DJ, Tao FB, Chen Q, et al. Incidence of injuries among rural boarding school students in Anhui province. *Chin J Sch Health*, 2008 , *29 (9)* :782-784.

89. Cao RX, Er YL, Zhang J. Epidemiological analysis of student injury in Haidian and pinggu district of Beijing city. *Chinese Journal of Health Education*, 2008 , *24 (11)* :822-824.

90. Lujiazui Sub-district. Analysis of epidemiological characteristics and influencing factors in Lujiazui Community. *The 5th Asian Regional conference on Safe Communities*, 2009.

91. Zhou WG, Jiang YQ, Guan LJ, et al. Researches about the prevalence of pre-school children's injuries and the status of their parents/guardians' kap in a kindergarten, shanghai. *China Prac Med*, 2009 ,*04 (20)* :257-259.

92. Luo Y, Tao FB, Zhang AnH. Injury incidence among school children in Bengbu city. *Chin J Public Health*, 2009 , *25 (8)* :926-927.

93. Wu XH, Wang HT, Chen JK. A study on incidence of injuries and its risk factors among teenagers in Nanxiong. *South China J Prev Med*, 2009 (5) :38-41.

94. Liang YF, Zhu D. Researches about unintentional injuries among pre-school children and the cognitive status of their mothers in a community. *Guangxi Medical Journal*, 2009 , *31 (3)* :423-425.

95. Dang XH, Xie H. Analysis on the incidence of injury and related risk behaviors among students in middle school of Xinyi. *Chin J School Doctor*, 2009 , *23 (2)* :192-193.

96. Zhang YY, Zheng HF, Wang FY. Investigation on accidental injuries of the students in several primary and secondary schools in Lanzhou. *Chinese Journal of Health Education*, 2009 , *25 (3)* :178-180.

97. Shen M. Epidemiological characteristics of unintentional injuries and evaluation of the intervention model for children in rural China. Diss, *Huazhong University of Science and Technology*, 2009..

98. Peng ZhQ, Zhou L, Dong GY. A study on incidence of injuries and risk factors among teenagers in Shenzhen. *Chinese Journal of Social Medicine*, 2009 , 26 (3) :168-171.

99. Zhao KF. A study on unintentional injury and mental health status of stay-at-home children in rural area of Anhui province. Diss, *Anhui Medical University*, 2009.

100. Li ShH. Study on epidemiological characteristics and influencing factors of unintentional injuries among children in PuCheng county. Diss, *Anhui Medical University*, 2009.

101. Li HW. An epidemiological prevalence study of injuries and related factors among elementary and middle school students in Haizhu district of Guangzhou. Diss, *Sun Yat-sen University*, 2009.

102. Du QW, Lu W, Liu Y, et al. Epidemiological survey on injuries among primary and middle school students in Ningyang county, 2007. *Chin J Sch Health*, 2009 , *30 (6)* :573-574.

103. He GL, Liu Y, Wu XJ, et al. Investigation on the unexpected injuries in children aged 1 to 14 years in Zhenjiang. *Modern Preventive Medicine*, 2009 , *36 (16)* :3012-3013.

104. Xia SH, Yu JL, Jin LJ. The analysis of accidental injury and risk factors about primary and secondary school students in Weifang. *Chin J Pest Contro*, 2009 (7) :509-511.

105. Liu W. Analysis on incidence of injury and its risk factors among primary and secondary school students in Guangzhou. Diss, *Jinan university*, 2009.

106. Zhang ChM, Liu YH, Jing ChD, et al. Investigation on the status of child injuries and needs of safety education in Tianjin. *Chinese Journal of Health Education*, 2010, *26 (2)* :98-100.

107. Fang YW, Zhu HL, Duan JW. Effectiveness evaluation on health education of unintentional injury for infants at Dinghai Kindergarten in Zhoushan City, Zhejang Province. *Chinese Journal of Health Education*, 2010 , *26 (3)* :225-226.

108. Hu YP. Community based cross-sectional study on injuries in Shanghai. Diss, *Fudan University*, 2010.

109. Liu XJ, Li LP, Zhao D, et al. Injury among children of 0 - 18 years old in island county, Guangdong province. *Chin J Public Health*, 2010 , *26 (12)* :1493-1494.

110. Mao ShJ, Huang XM, Yang YH. Investigation on injuries among children aged 0~6 and the cognitive behavior of their parents. *Zhenjiang Preventive Medicine*, 2010 ,*22 (5)* :80-81.

111. Xu W, Wu YSh, Wan QP, et al. Analyze of epidemiological characteristics of community injuries in Zhabei district, Shanghai. *Disease Surveillance*, 2010 , *25 (5)* :417-419.

112. Su YAn, Sun RR, Huang ShG. Investigation on the incidence of accidental injury and related risk behaviors among primary school students in rural areas of Yingde. *Chin. J of PHM*, 2010 (2) :198-200.

113. Wang KR, Ren BB, Wen J, et al. Study on characteristics of injuries among students in middle and primary schools of Yinchuan. *Journal of Hygiene Research*, 2010 , *39 (3)* :352-354.

114. Chen N. Study on situation and influencing factors of injuries in urban primary and junior high school students. Diss, *Shandong University*, 2010.

115. Zhou YF, Luo ChY, Lu X. Epidemiological Survey of Injuries Among Primary and Secondary School Students in Shanghai. *Chin J Sch Health*, 2010 , *31 (11)* :1369-1372.

116. Yan WJ, Yan H. Analysis on the incidence of accidental injuries among left-behind children of Bai in mountain areas of Dali. *Chinese Community Doctors*, 2011 ,*13 (12)* :266-266.

117. Lin M, Bai Y, Shi P, et al. A survey on injury and its related behaviors among middle school students in Shijingshan district, Beijing. *Capital Journal of Public Health*, 2011 ,*05 (4)* :164-167.

118. Shi XY, Yang LL, Jin R, et al. Investigation on accidental injuries of junior high school students in Wenzhou. *Nurs J Chin PLA*, 2011 , *28 (21)* :9-11.

119. Shen Y, Zhang YY, Zhang Q, et al. Retrospective investigation on incidence of accidental injury among students in middle and primary school of Jiading district, Shanghai. 2011 , 25 (6) :110-112.

120. A study on the incidence of accidental injuries and risk factors among primary school students in Xinzhuang of Minhang district. *World Health Digest Medical Periodieal*, 2011 , *08 (22)* :133-136.

121. Shi ZhF, Chen N, Zhao ZhT. Study on injury status and related factors among urban pupils in Ji' nan City, 2008. 2011 (7) :598-599.

122. Jiang W, Huang HX, Wei JL, et al. Epidemiological investigation on unintentional injuries of left-behind children in rural area of Nanning. *Maternal and Child Health Care of China*, 2011 ,*26 (4)* :563-565.

123. Jiang W, Pan ChH, Liu W, et al. Epidemiological study on accidental injuries among preschool children in Nanning. *CJCHC*, 2011 , *19 (1)* :24-26.

124. Zhang JW, Zhang J, Lu HZh, et al. Analysis of epidemiology of injuries among primary and middle school students in Qingpu District. *Chinese Journal of General Practice*, 2011 , *09 (4)* :606-608.

125. Wang P, Wei L, Qin YX, et al. Analysis on epidemiological feature of unintentional injuries in primary and middle school students of Liuzhou. *Modern Preventive Medicine*, 2011,*38(03)*:401-403+408.

126. Zhu XX, Liu QM, Fang ShY, et al. Epidemic characters of injuries among school aged children of migrant workers in Hangzhou. *Chin J Sch Health*, 2011 ,*32 (2)* :203-205.

127. Wei L, Huang YQ, Shi NN. Risk factors for accidental injuries among middle and primary schools students in Jiangbei district of Ningbo. *Chin J Sch Health*, 2011 , *32 (6)* :741-742.

128. Xie XH, Fan ZhY, Tan ZW, et al. Analysis of preschool-age year-old child injury in Haidian and Pinggu area of Beijing. *Chin Pediatr Emerg Med*, 2011, *18 (3)* :226-229.

129. Hu GQ, Zhu SL, Wang QQ, et al. An epidemiological survey on the incidence of non-fatal injury and influencing factors among children under 5 years old in China. *Chin J Epidemiol*, 2011 , *32 (8)* :773-776.

130. Liu WL, Dou WJ, Chu WJ, et al. Survey on injuries among rural residents in Shandong province. *Prev Med Trib*, 2009~2010. 2012 *18 (3)* :187-190.

131. Wu MY, Zhang Y, Li Q. An epidemiological survey on injuries among preschool children in Huangpu district of Shanghai. *Chin J of PHM*, 2012 ,*28 (6)* :800-801.

132. Shi RX, Wang H, Lu R, et al. A study on the incidence of severe injuries and risk factors among students in middle schools in Fengtai district, Beijing. *Chin J Sch Health*, 2012,*33(09)*:1145-1147.

133. Wu XH, Dai ShG, Dai ChY, et al. Sheyang county 2010 prevalence study on injuries among students. *Chinese Primary Health Care*, 2012 , *26 (12)* :29-31.

134. Tang HY, Lu Y. Injury epidemiology analysis of primary and middle school students in Fengxain district, Shanghai. *Health Education and Health Promotion*, 2012 (1) :29-32.

135. Sun Y. The characteristics of injury proneness and its influencing factors among children in one rural area of Anhui Province. Diss, *Anhui Medical University*, 2012.

136. Yang DY, Wang YH, Chen TR. Injury incidence and disease burden analysis on primary and middle schools in Cangnan County. *Zhejiang Preventive Medicine*, 2012 ,*24 (7)* :11-14.

137. Zhang L, Dai X. Prevalence and risk factors of injury among children aged 0~14 years in Hubei province. *Chin J Sch Health*, 2012 , *33 (9)* :1111-1113.

138. Hu M, Hu GQ, Sun ZhQ, et al. Epidemiological Survey of the Prevalence of Non-fatal Injury among Children Aged 5-14 Years in China. *Biomed Environ Sci*, 2012, *25(4)*:407-412.

139. Yang J, Li Y, Zhang SH, et al. Study on the incidence of injury and analysis on the influencing factors among middle school students in Haizhu District, Guangzhou City. *Chin J Dis Control Prev*, 2013 ,*17 (7)* :588-591.

140. Sun N, Ren XM, Bai YF. The survey on the parents′awareness and knowledge about the accidental injuries in children in Xianyang City. *Journal of Xinjiang Medical University*, 2013 (10) :1523-1525.

141. Wang XX, Zhao Y. Survey on status of injuries among students in middle and primary school of Dalian. *China Health Care and Nutrition*,  2013 (11).

142. Li GC, Yang GM. Analysis of the investigation of the accident injuries in children aged under six years old in three towns and nine villages of Luquan county. *Medicine and Pharmacy of Yunnan*, 2013 (1) :60-61.

143. Yao YH, Yao W, Chen DY, et al. Epidemic characteristics of injuries among residents of Hongkou District. *J Environ Occup Med*, 2013 , *30 (11)* :863-866.

144. Zhang MR, Tian R, Yang Zh, et al. An epidemiological investigation on injuries among community residents in Kunming. *Chin J Prev Contr Chron Dis*, 2013 , *21 (1)* :56-57.

145. Zhou Y. Unintentional injuries and their influencing factors among middle school students in Bengbu City, Anhui Province. Diss, *Bengbu Medical College*, 2013.

146. Hu MJ, Ma WJ, Xu YJ, et al. Survey on injury spectrum and economic burden of injuries in residents of Guangdong Province. *South China J Prev Med*, 2013 (1) :18-22.

147. He J, Xu ShX, Yang B, et al. Analysis on the risk factor of injuries for primary and middle school students in Qujing city. *Chin J Prev Contr Chron Dis*, 2013 , *21 (5)* :565-567.

148. Shao QJ, Sun X. Investigation on accidental injuries among middle school and under students in county of Henan. *Henan Medical Research*, 2014 (9) :144-147.

149. Jia GZh, Song LD, Xu TH, et al. Study on epidemiological characteristics and influencing factors of unintentional injuries among rural children in Dongming county, Heze city. *CJCHC,* 2014 , *22 (4)* :423-426.

150. Zhao LF, Su PY. Unintentional injuries and its influencing factors among adolescents in rural areas. *China Health Care Nutrition*, 2014(6).

151. Yang YL, Xue L, Cui LH, et al. Investigation and analysis of accidental injury of 3~6 years old rural children in Zibo County. *CJCHC*, 2014 ,*22 (1)* :93-95.

152. Zhang XJ, Gao H, Guo ChY, et al. An investigation on injuries among pupils of 6 primary school in Ningbo City. *Chinese Journal of Health Education*, 2014 , *30 (4)* :356-359.

153. Yang YC, Wang LR, Gao LH. A cross-sectional study on injuries among students in middle schools of Zhengzhou. *J of Pub Health and Prev Med*, 2014 , *25 (5)* :115-117.

154. Wei YH, Wang DH, Jing QL, et al. Epidemiological analysis of primary students unintentional injury in Guangzhou area. *J Med Pest Control*, 2014, *30 (7)* :711-714.

155. Zhang YX, Yang J, Chen WY, et al. Analysis on incident injuries and related risk factors among adolescents in Changzhou. *Jiangsu J Prev Med*, 2014 , *25 (4)* :32-34.

156. Deng FM, Gong XM, Cui HY, et al. Risk factors for unintentional injury among children in rural areas of Liling, Hunan Province, China. *Chin J Contemp Pediatr*,  2014 ,*16 (5)* :524-528.

157. Duan JL, Fu Y, Lv Y. Analysis on the status and consequences of injury among primary and middle school students in Beijing. *Chin J Sch Health*, 2014 , *35 (6)* :938-939.

158. Nie ShP, Shen ShJ, Mai ZhH, et al. Prevalence and risk factors of injuries among middle school students in Guangdong Province. *Chin J Sch Health*, 2014 , *35 (4)* :493-496.

159. Zhang GZh, Ma JF, Yao XM. Research of risk factors for accidental injuries in preschool children of Urumqi City. *China Health Industry*,  2015 (4) :7-8.

160. Zhang JB, Du ZhQ, Fu YG. An epidemiological survey on injury among the international security community residents in Changzhi. *Modern Preventive Medicine*, 2015 ,*42 (16)* :2949-2952.

161. Dou DM, Wang PX. Correspondence analysis of association between types of unintentional injuries and influential factors among rural rear pupils. *Journal of Hygiene Research*, 2015 , *44 (4)* :549-552。

162. Liu HY, Yang L, Feng Q, et al. Epidemiological characteristics and risk factors of the unintentional injuries among migrant children aged 3~6 years old in Guangzhou. *CJCHC*, 2015 , *23 (4)* :419-421.

163. Wang Li, Mao X, Hao ZhH, et al. Parental rearing pattern and child injury proneness among grade 4~5 students in Taiyuan. *Chin J Sch Health*, 2015 ,*36 (9)* :1355-1357.

164. Zhang H, Li Y, Cui YX, et al. The study on the relationship of incidence of injuries among children and the cognition of their parents in Daqing. *Chin J Sch Health*, 2015 , *36 (3)* :427-429.

165. Zhang ShL, Yao SP, Guo Y, et al. Epidemiology of injuries for middle school students, Jiamusi City. *Modern Preventive Medicine*, 2015 , *42 (13)* :2316-2318.

166. Qu ShX, Wang ShM, Zheng WJ, et al. Injury of pre-school children and related factors in a community of Shanghai. *Chin Prev Med*, 2015 , *16 (3)* :191-195.

167. Luo J. Study on prevalence, influence factors and evaluation of educational intervention effect of injury among the school-aged children in rural area of Hanchuan. Diss, *Wuhan University of Science and Technology*, 2015.

168. Guo L, Zhang ShH, Shao HY, et al. Study on the risk behaviors related to injury of adolescent in Anyang, 2013. *Chin J Sch Health*, 2015 ,*36 (7)* :1109-1111.

169. Tao X, Xiang B, Luo J, et al. Study on the current situation and risk factors of injuries among school children in rural area of Enshi. *Maternal and Child Health Care of China*, 2015, *30 (32)* :5637-5639.

170. Fang Y, Zhang X, Chen W, et al. Epidemiological characteristics and burden of childhood and adolescent injuries: a survey of elementary and secondary students in Xiamen, China. *Bmc Public Health*, 2015, *15(1)*:357.

171. Research on epidemiological characteristics and influencing factors of unintentional injuries among left-behind children in poverty areas in Yunnan. Diss, *Kunming Medical University*, 2015.

172. Zhang HZh. A study on incidence of injuries and influencing factors among middle school students in Yuhong district of Shenyang. *Chin J Sch Health*, 2015 , *36 (5)* :782-784.

173. Sun QL, Gao F, Liu XF, et al. Study on injuries and evaluation of the intervention among primary school students in Bao’an district of Shenzhen. *Chin J Sch Health*, 2015 , *36 (5)* :777-779.

174. Peng ZhH, Wang H, Shi YP, et al. Study on prevalence and household - environmental factors of child injury in kindergartens of Shenzhen city. *Maternal and Child Health Care of China*, 2015 ,*30 (36)* :6569-6571.

175. Liu XX, Gao HM, Ye KY, et al. Incidence and inﬂuencing factors of child injury in schools for migrant worker’s children in Qingpu District of Shanghai in 2015. *J Environ Occup Med*, 2016 , *33 (11)* :1093-1096.

176. Guo Q, Xiang B, Tao X, et al. Analysis on factors of injuries among left-behind junior high school students in rural areas of Enshi. *Injury Medicine(Electronic Edition)*, 2016 , *6 (2)* :5-9.

177. Yang XW, Cha WT, Zhang GCh, et al. Multilevel model study on status and influencing factors of accident injury among rural school-age children in Hunan Province. *Chin J Dis Control Prev* , 2016 , 20 (3) :266-270.

178. Liu X, Zhang YB, Zhang GH, et al. Survey and study on unintentional injuries of rural left -behind children of Jining City. *China Journal of Health Psychology*, 2016 (1) :78-80.

179. Chang YH, Fan S, Chen H, et al. Injury among junior high school students in Luzhou city. *Chin J Sch Health*, 2016 ,*37 (11)* :1666-1669.

180. Sun GX, Cao L, Tao LL, et al. A study on incidence of injuries and influencing factors among rural students in middle schools of a county. *J Med Theor & Prac*, 2016 ,*29 (23)* :3290-3292.

181. Hao ZhH. The study of the unintentional injury and the relationship with parental rearing pattern in school children. Diss, *Shanxi Medical University*, 2016.

182. Sheng JH. Study on status and risk factors of unintentional injuries among students in primary in rural areas of Yunnan Province. Diss, *Kunming Medical University*, 2016.

183. Huang Y, Li XM, Tang M, et al. Influencing factors of unintentional injuries among the left-behind children in a poverty county in Yunnan Province. *Journal of Kunming Medical University*, 2016 , *37 (12)* :37-40.

184. Lian XX, Zuo QH, Ran YCh. 3-6 years old children’s injuries in Aba Tibetan and Qiang autonomous prefecture,2014. *Modern Preventive Medicine,* 2017,*44(15)*:2748-2750+2764.

185. Ren YP, Shen HP, Bai PQ, et al. Epidemiological characteristics and risk factors of injury among primary school students in Pudong New Area, Shanghai. *Chin J Sch Health*, 2017 , *38 (11)* :1688-1690.

186. Zhu J, Long LH, Cao ShH, et al. Epidemiological study on unintentional injuries in children aged 0-6 years in Shaoguan District. *China Modern Medicine,* 2017 ,*24 (29)* :137-139.

187. Zhang XZh, Chen L, Liu GY. A study on the incidence and risk factors of injury among primary school students in Wenzhou. *Chin J Prev Contr Chron Dis,* 2017 ,*25 (4)* :277-280.

Table S1. Characteristics of included studies among children and adolescents(aged 0-19 years)

| First author/Publi-  cation year (study  period | Study site  (region) | Version of injury definition | Age | Sample size | Number of injured person | Incidence rate  (%) | Incidence rate in boys | Incidence rate in girls | Incidence rate in urban | Incidence rate in rural | Quality score |
| --- | --- | --- | --- | --- | --- | --- | --- | --- | --- | --- | --- |
| Wang JX/2000  (1999) | Handan city  (eastern region) | Version of 1996 | Middle School  students | 785 | 291 | 37.07 | — | — | — | — | 5 |
| Hu LR/2000  (—) | Zhanjiang city  (eastern region) | Version of 1996 | Middle School  students | 1969 | 1572 | 79.84 | 873/1086 | 699/883 | — | — | 5 |
| Han YX/2000  (1998) | Handan city  (eastern region) | Version of 1996 | Primary and middle school students | 10449 | 1131 | 10.82 | — | — | — | — | 6 |
| Wang ShY/2000  (1999) | Guangdong province  (eastern region) | Version of 1996 | Primary and middle school students | 14533 | 6941 | 47.76 | 3945/7878 | 2996/6655 | — | — | 5 |
| Wu KM/2001  (1999) | Chengdu city  (western region) | Version of 1996 | Kindergarten children | 2165 | 634 | 29.28 | 370/1146 | 264/1019 | 634/2165 | — | 7 |
| Liu L/2001  (1999) | Guiyang city  (western region) | Version of 1996 | Kindergarten children | 2193 | 543 | 24.76 | 314/1179 | 229/1014 | 543/2193 | — | 6 |
| Yang BH/2001  (2000) | Chenzhou city  (middle region) | Version of 1996 | Primary and middle school students | 3104 | 2332 | 75.13 | 1351/1648 | 981/1456 | — | — | 7 |
| Ye DQ/2001  (2000) | Huaiyuan county  (middle region) | Version of 1996 | Primary and middle school students | 15149 | 5911 | 39.02 | 3560/8465 | 2351/6684 | — | — | 6 |
| Li YZh/2002  (1999) | Tongjiang County  (western region) | Version of 1996 | 0-14 years old | 324 | 116 | 35.80 | — | — | — | 116/324 | 4 |
| Xu YQ/2002  (2001) | Shiyan City  (middle region) | Version of 1996 | 0-6 years old | 527 | 156 | 29.60 | 108/297 | 48/230 | — | 156/527 | 4 |
| Ni Y/2002  (2000) | Shantou city  (eastern region) | Version of 1996 | Kindergarten children | 1435 | 192 | 13.38 | 105/719 | 86/699 | 192/1435 | — | 6 |
| Huang WQ/2002  (2001) | Wuhan city  (middle region) | Version of 1996 | Primary and middle school students | 1703 | 502 | 29.48 | 294/883 | 208/820 | — | — | 6 |
| Yao YSh/2002  (2000) | Anhui south region  (middle region) | Version of 1996 | middle school students | 2135 | 1431 | 67.03 | 896/1255 | 535/880 | — | — | 7 |
| Liang HY/2002  (2000) | Shenzhen city  (eastern region) | Version of 1996 | Primary and middle school students | 2181 | 340 | 15.59 | 210/1180 | 130/1001 | 340/2181 | — | 6 |
| Hu ShF/2002  (2001) | PingLiang city  (western region) | Version of 1996 | Primary and middle school students | 4820 | 2287 | 47.45 | — | — | 1194/2400 | 1093/2420 | 5 |
| Zhao JH/2002  (2000) | Ningxia  (western region) | Version of 1996 | Primary and middle school students | 6766 | 1380 | 20.40 | 816/3434 | 564/3332 | — | — | 6 |
| Li AL/2003  (2001) | Hangzhou city  (eastern region) | Version of 1996 | Primary school students | 964 | 558 | 57.88 | 308/488 | 250/476 | 558/964 | — | 5 |
| Liu XL/2003  (2000) | Jinzhou city /Beining city  (eastern region) | Version of 1996 | Primary school students | 1153 | 410 | 35.56 | — | — | — | — | 6 |
| Fu M/2003  (2003) | Zhuhai city  (eastern region) | Version of 1996 | Kindergarten children | 1308 | 714 | 54.59 | 433/745 | 281/563 | 714/1308 | — | 6 |
| Zhao JB/2003  (2002) | Harbin  (middle region) | Version of 1996 | Primary school students | 1629 | 449 | 27.56 | 240/804 | 209/825 | 449/1629 | — | 5 |
| Liu WG/2003  (2000) | Yinchuan City  (western region) | Version of 1996 | Primary school students | 1760 | 645 | 36.65 | 382/915 | 263/845 | — | — | 4 |
| Zhao JB/2003  (2002) | Harbin  (middle region) | Version of 1996 | middle school students | 2388 | 686 | 28.73 | 406/1181 | 280/1207 | — | — | 5 |
| Wang YZh/2003  (—) | Xining city  (eastern region) | Version of 1996 | middle school students | 2427 | 1085 | 44.71 | 342/682 | 743/1745 | — | — | 4 |
| Shi MC/2003  (2001) | Xinjiang  (western region) | Version of 1996 | Kindergarten children/Primary school students | 2498 | 878 | 35.15 | 493/1314 | 385/1184 | 253/622 | 625/1876 | 6 |
| Li LP/2003  (1997) | Shantou city  (eastern region) | Version of 1996 | Primary and middle school students | 2553 | 969 | 37.96 | 545/1324 | 424/1229 | — | — | 7 |
| Wang J/2003  (2001) | Jiuquan city  (western region) | Version of 1996 | Primary and middle school students | 8507 | 3600 | 42.32 | 1954/4329 | 1646/4178 | — | — | 6 |
| Liu AM/2003  (2002) | Dafeng city  (eastern region) | Version of 1996 | Primary and middle school students | 9525 | 3564 | 37.42 | — | — | — | — | 5 |
| Xing GY/2003  (2002) | Linyi city  (eastern region) | Version of 1996 | Primary and middle school students | 11349 | 4721 | 41.6 | 2746/6092 | 1975/5257 | — | — | 6 |
| Huang F/2003  (2001) | Suzhou city  (middle region) | Version of 1996 | Primary and middle school students | 15642 | 3810 | 24.36 | 2226/8389 | 1584/7253 | — | 3810/15642 | 6 |
| Zhong JM/2003  (2001) | Zhejiang province  (eastern region) | Version of 1996 | Primary and middle school students | 42065 | 7454 | 17.72 | — | — | — | — | 5 |
| Ye Y/2004  (2003) | Wuhan city  (middle region) | Version of 1996 | middle school students | 584 | 117 | 20.03 | 64/281 | 50/296 | — | — | 6 |
| Sun Y/2004  (2003) | Linyi city  (eastern region) | Version of 1996 | Primary school students | 964 | 558 | 57.88 | 308/488 | 250/476 | 558/964 | — | 5 |
| Pang ShL/2004  (2002) | Tangshan city  (eastern region) | Version of 1996 | middle school students | 1288 | 572 | 44.41 | — | — | — | — | 5 |
| Wu XH/2004  (2000) | Huangshi city  (middle region) | Version of 1996 | Primary school students | 1748 | 491 | 28.09 | 270/922 | 221/826 | 491/1748 | — | 4 |
| Shi Y/2004  (2003) | Beijing city  (eastern region) | Version of 1996 | 0-14 years old | 2391 | 319 | 13.34 | — | — | 157/1220 | 162/1171 | 5 |
| Lin Y/2004  (2001) | Shenzhen city  (eastern region) | Version of 1996 | 0-6 years old | 3750 | 122 | 3.25 | 72/2097 | 50/1653 | 122/3750 | — | 5 |
| Dai LP/2004  (2003) | Loudi city  (middle region) | Version of 1996 | Primary school students | 3830 | 837 | 21.85 | — | — | 396/2062 | 441/1768 | 5 |
| Zhang Y/2004  (2002) | Ma’anshan city  (middle region) | Version of 1996 | Primary school students | 4043 | 657 | 16.25 | — | — | — | — | 5 |
| Xiao L/2004  (2003) | Nanjing city  (eastern region) | Version of 1996 | Primary and middle school students | 4067 | 1682 | 41.36 | 938/2076 | 744/1991 | — | — | 5 |
| Li ZhH/2004  (2003) | Dongying city  (eastern region) | Version of 1996 | 0-19 years old | 4137 | 207 | 5 | — | — | — | 207/4137 | 7 |
| Chang JX/2004  (2003) | Rongcheng city  (eastern region) | Version of 1996 | Primary and middle school students | 4361 | 2217 | 50.84 | 1154/2207 | 1063/2154 | — | — | 5 |
| Zhang Y/2004  (2002) | Ma’anshan city  (middle region) | Version of 1996 | Primary school students | 6884 | 1710 | 24.84 | — | — | 1710/6884 | — | 7 |
| Xu ShD/2005  (2005) | Wuhan city  (middle region) | Version of 1996 | Kindergarten children | 822 | 192 | 23.36 | 118/491 | 74/331 | 192/822 | — | 4 |
| Zhang D/2005  (2004) | Beijing city  (eastern region) | Version of 1996 | Primary and middle school students | 855 | 108 | 12.63 | 63/446 | 45/409 | — | — | 4 |
| Wang H/2005  (2002) | Shenzhen city  (eastern region) | Version of 1996 | 0-14 years old | 897 | 75 | 8.36 | 51/493 | 24/404 | — | — | 4 |
| He ZhH/2005  (2003) | Guangzhou city  (eastern region) | Version of 1996 | 0-14years old | 1285 | 210 | 16.34 | 118/648 | 92/637 | — | — | 6 |
| Li SF/2005  (2002) | Guangzhou city  (eastern region) | Version of 1996 | 0-14 years old | 1354 | 112 | 8.27 | — | — | 112/1354 | — | 6 |
| Wei Y/2005  (2004) | Jining city  (eastern region) | Version of 1996 | Primary and middle school students | 1554 | 543 | 34.94 | 351/894 | 192/660 | — | 543/1554 | 6 |
| Zhang YF/2005  (2003) | Shaoxing city  (eastern region) | Version of 1996 | 0-14 years old | 2128 | 354 | 16.64 | 215/1085 | 139/1043 | — | — | 7 |
| Chu JF/2005  (2003) | Hangzhou city  (eastern region) | Version of 1996 | Primary and middle school students | 2631 | 819 | 31.13 | 495/1370 | 325/1261 | — | 819/2631 | 6 |
| Liu L/2005  (2002) | Yinchuan city  (western region) | Version of 1996 | Primary and middle school students | 4595 | 2670 | 58.11 | — | — | — | — | 6 |
| Xu GZh/2005  (-) | Ningbo city  (eastern region) | Version of 1996 | middle school students | 5612 | 1204 | 21.45 | — | — | — | — | 6 |
| Chen X/2005  (2002) | Xuzhou city  (eastern region) | Version of 1996 | 0-14 years old | 6085 | 1217 | 20 | 716/3238 | 501/2847 | 1217/6085 | — | 5 |
| Yan XY/2006  (2005) | Yueyang city  (middle region) | Version of 1996 | Kindergarten children | 456 | 126 | 27.63 | 68/234 | 58/222 | 126/456 | — | 6 |
| Xiong JF/2006  (2004) | Shenzhen city  (eastern region) | Version of 1996 | Primary and middle school students | 483 | 127 | 26.29 | — | — | — | — | 5 |
| Xu HQ/2006  (2005) | Guangzhou city  (eastern region) | Version of 1996 | Primary and middle school students | 600 | 165 | 27.5 | 101/298 | 64/302 | 165/600 | — | 5 |
| Xu W/2006  (2004) | Shanghai city  (eastern region) | Version of 1996 | 0-14 years old | 650 | 15 | 2.31 | — | — | — | — | 6 |
| Ma DH/2006  (2004) | Yanqing County  (eastern region) | Version of 1996 | Primary and middle school students | 821 | 116 | 14.13 | 68/355 | 48/466 | — | — | 6 |
| Shao YQ/2006  (2004) | Shanghai city  (eastern region) | Version of 1996 | Primary and middle school students | 976 | 103 | 10.55 | 73/584 | 30/392 | — | — | 5 |
| Zhang Y/2006  (2005) | Jinan city  (eastern region) | Version of 1996 | middle school students | 1879 | 847 | 45.08 | 146/281 | 701/1598 | — | — | 4 |
| Yu Y/2006  (2004) | Nanjing city  (eastern region) | Version of 1996 | Primary and middle school students | 2016 | 593 | 29.41 | 353/1104 | 240/912 | — | — | 6 |
| Zhou DD/2006  (2003) | Shanghai city  (eastern region) | Version of 1996 | 0-14 years old | 2695 | 286 | 10.61 | 145/1331 | 141/1364 | 144/1375 | 142/1320 | 6 |
| Chu QP/2006  (2004) | Xuzhou city  (eastern region) | Version of 1996 | middle school students | 2990 | 914 | 30.57 | 464/1258 | 450/1732 | — | — | 6 |
| Tan ShK/2006  (2003) | Baise area  (western region) | Version of 1996 | Primary and middle school students | 3523 | 750 | 21.29 | — | — | — | — | 6 |
| Nie ShP/2006  (2005) | Guangdong province  (eastern region) | Version of 2004 | middle school students | 17319 | 6167 | 35.61 | 3318/8755 | 2849/8564 | — | — | 6 |
| Zhao D/2007  (2006) | Nan'ao County  (eastern region) | Version of 1996 | 0-15 years old | 329 | 153 | 46.5 | — | — | — | — | 5 |
| Xu WM/2007  (2006) | Hangzhou city  (eastern region) | Version of 1996 | Primary school students | 436 | 241 | 55.28 | 151/247 | 90/189 | — | — | 4 |
| Li LJ/2007  (2004) | Wuhan city  (middle region) | Version of 1996 | 0-10 years old | 720 | 211 | 29.31 | 149/398 | 62/322 | — | — | 4 |
| Yang LX/2007  (2004) | Beijing city  (eastern region) | Version of 1996 | Primary and middle school students | 840 | 71 | 8.45 | 35/362 | 36/478 | — | — | 6 |
| Ding YP/2007  (2004) | Beijing city  (eastern region) | Version of 1996 | Primary and middle school students | 841 | 59 | 7.02 | 37/399 | 22/442 | — | — | 4 |
| Cai LG/2007  (2005) | Beijing city  (eastern region) | Version of 1996 | Primary school students | 935 | 582 | 62.25 | 325/485 | 257/450 | — | — | 5 |
| Wang RSh/2007  (-) | Wendeng city  (eastern region) | Version of 1996 | Primary school students | 1180 | 537 | 45.51 | 305/620 | 232/560 | — | — | 4 |
| Xue L/2007  (2006) | Tangshan city  (eastern region) | Version of 1996 | Primary school students | 1223 | 588 | 48.08 | 260/507 | 328/716 | — | 588/1223 | 5 |
| Xiang B/2007  (2004) | Wuhan city  (middle region) | Version of 1996 | Kindergarten children | 1690 | 252 | 14.91 | — | — | — | — | 6 |
| Zhang YY/2007  (2005) | Hezuo city  (western region) | Version of 1996 | Primary school students | 1964 | 727 | 37.02 | — | — | — | — | 5 |
| Xing YP/2007  (2006) | Shangqiu City  (middle region) | Version of 1996 | middle school students | 2038 | 576 | 28.26 | 336/984 | 240/1054 | — | — | 6 |
| Yao YSh/2007  (2006) | anhui south region  (middle region) | Version of 1996 | 3-6 years old | 2385 | 751 | 31.49 | 467/1245 | 284/1140 | — | — | 6 |
| Wang YL/2007  (2005) | Shanghai city  (eastern region) | Version of 1996 | Primary school students | 2610 | 332 | 12.72 | 181/1281 | 151/1329 | — | — | 6 |
| Li N/2007  (2004) | Liaoning province  (eastern region) | Version of 1996 | 0-18 years old | 3644 | 604 | 16.58 | — | — | — | — | 5 |
| Yang YM/2007  (2005) | South Jiangsu  (eastern region) | Version of 1996 | 0-18years old | 22126 | 3310 | 14.96 | 1919/11207 | 1391/10919 | — | — | 7 |
| Zhu LP/2007  (2005) | Jiangxi province  (middle region) | Version of 2004 | 0-17years old | 98335 | 5567 | 5.66 | 3815/55365 | 1752/42970 | 1034/17480 | 4533/80855 | 7 |
| Li SS/2008  (2006) | Shanghai city  (eastern region) | Version of 2004 | 0-14 years old | 470 | 5 | 1.06 | — | — | 5/470 | — | 7 |
| Li L/2008  (2007) | Jinan city  (eastern region) | Version of 1996 | 4-6 years old | 873 | 164 | 18.79 | 109/461 | 55/412 | 164/873 | — | 6 |
| Wu DN/2008  (2007) | Shenzhen city  (eastern region) | Version of 2004 | middle school students | 1011 | 435 | 43.03 | 258/503 | 177/508 | — | — | 6 |
| Zhang XP/2008  (2008) | Jiujiang city  (middle region) | Version of 1996 | middle school students | 2500 | 1281 | 51.24 | 868/1445 | 413/1055 | — | — | 5 |
| Zeng ChJ/2008  (2007) | Shiyan city  (middle region) | Version of 1996 | Primary school students | 2955 | 146 | 4.94 | 103/1547 | 43/1408 | 146/2955 | — | 4 |
| Xu ZN/2008  (2006) | Qidong city  (eastern region) | Version of 2004 | middle school students | 4051 | 1283 | 31.67 | — | — | — | — | 6 |
| Cheng DJ/2008  (2008) | Anhui province  (middle region) | Version of 1996 | Primary and middle school students | 5556 | 2891 | 52.03 | — | — | — | 2891/5556 | 5 |
| Cao RX/2008  (2004) | Beijing city  (eastern region) | Version of 2004 | Primary and middle school students | 17875 | 1361 | 7.61 | — | — | 853/10331 | 508/7544 | 7 |
| Lujiazui street office/2009  (2008) | Shanghai city  (eastern region) | Version of 2004 | 0-14 years old | 195 | 3 | 1.54 | — | — | 3/195 | — | 6 |
| Zhou WG/2009  (2008) | Shanghai city  (eastern region) | Version of 1996 | Kindergarten children | 227 | 29 | 12.78 | — | — | 29/227 | — | 4 |
| Luo Y/2009  (2007) | bengbu city  (middle region) | Version of 1996 | Primary and middle school students | 738 | 272 | 36.86 | 138/349 | 134/389 | — | — | 5 |
| Wu XH/2009  (2007) | Nanxiong city  (eastern region) | Version of 2004 | middle school students | 871 | 437 | 50.17 | — | — | — | 437/871 | 6 |
| Liang YF/2009  (2006) | Nanning city  (western region) | Version of 1996 | 2-6 years old | 951 | 422 | 44.37 | 245/532 | 177/419 | 422/951 | — | 5 |
| Dang XH/2009  (2008) | Xinyi city  (eastern region) | Version of 2004 | middle school students | 1027 | 120 | 11.68 | 69/413 | 51/614 | — | — | 6 |
| Zhang YY/2009  (2008) | Lanzhou city  (western region) | Version of 1996 | Primary and middle school students | 1612 | 458 | 28.41 | 312/871 | 146/741 | — | — | 5 |
| Shen M/2009  (2006) | Macheng city  (middle region) | Version of 1996 | Primary and middle school students | 3019 | 519 | 17.19 | 396/1821 | 123/1198 | — | 519/3019 | 6 |
| Zhao KF/2009  (-) | Changfeng county  (middle region) | Version of 2004 | Primary and middle school students | 3522 | 1136 | 32.25 | 687/1886 | 449/1636 | — | 1136/3522 | 7 |
| Peng ZhQ/2009  (2007) | Shenzhen city  (eastern region) | Version of 2004 | middle school students | 3526 | 908 | 25.75 | 584/1759 | 324/1767 | — | — | 6 |
| Li ShH/2009  (2008) | Pucheng county  (western region) | Version of 1996 | 0-14 years old | 3604 | 486 | 13.49 | — | — | — | 486/3604 | 7 |
| Li HW/2009  (2008) | Guangzhou city  (eastern region) | Version of 1996 | Primary and middle school students | 3677 | 1964 | 53.41 | 1067/1950 | 897/1727 | — | — | 8 |
| Du QW/2009  (2007) | Ningyang county  (eastern region) | Version of 1996 | Primary and middle school students | 5059 | 1983 | 39.2 | 1304/2634 | 679/2425 | — | — | 6 |
| He GL/2009  (2005) | Zhenjiang city  (eastern region) | Version of 1996 | 1-14years old | 5477 | 528 | 9.64 | 311/2641 | 217/2836 | — | — | 6 |
| Xia SH/2009  (1998) | Weifang City  (eastern region) | Version of 1996 | Primary and middle school students | 22384 | 1538 | 6.87 | 994/11678 | 544/10710 | — | — | 6 |
| Liu W/2009  (2008) | Guangzhou city  (eastern region) | Version of 1996 | Primary and middle school students | 24168 | 5227 | 21.63 | 2933/11609 | 2294/12559 | — | — | 7 |
| Zhang ChM/2010  (2009) | Tianjin city  (eastern region) | Version of 1996 | Primary and middle school students | 271 | 143 | 52.77 | 83/140 | 60/131 | — | — | 4 |
| Fang YW/2010  (2008) | Zhoushan city  (eastern region) | Version of 1996 | Kindergarten children | 273 | 166 | 60.81 | — | — | 166/273 | — | 4 |
| Hu YP/2010  (2008) | Shanghai city  (eastern region) | Version of 2004 | 0-14years old | 363 | 4 | 1.1 | — | — | — | — | 6 |
| Liu XJ/2010  (2006) | Island county  (eastern region) | Version of 1996 | 0-18years old | 452 | 228 | 50.44 | 142/236 | 86/216 |  |  | 4 |
| Xu W/2010  (2007) | Shanghai city  (eastern region) | Version of 1996 | 0-14years old | 480 | 5 | 1.04 | — | — | 5/480 | — | 6 |
| Mao ShJ/2010  (-) | Hangzhou city  (eastern region) | Version of 1996 | 0-6years old | 835 | 226 | 27.07 | — | — | — | — | 6 |
| Su YA/2010  (2009) | Yingde City  (eastern region) | Version of 1996 | Primary school students | 3050 | 693 | 22.72 | 403/1532 | 290/1518 | — | 693/3050 | 6 |
| Wang KR/2010  (2009) | Yinchuan City  (western region) | Version of 1996 | Primary and middle school students | 3489 | 570 | 16.34 | 299/1631 | 271/1858 | 270/1885 | 300/1604 | 7 |
| Chen N/2010  (2006) | Jinan city  (eastern region) | Version of 1996 | Primary and middle school students | 3714 | 763 | 20.54 | 385/1901 | 378/1813 | 763/3714 | — | 8 |
| Zhou YF/2010  (2009) | Shanghai city  (eastern region) | Version of 2004 | Primary and middle school students | 29416 | 1478 | 5.02 | — | — | — | — | 6 |
| Yan WJ/2011  (2010) | Dali city  (western region) | Version of 2004 | Primary school students | 78 | 33 | 42.31 | — | — | — | 33/78 | 4 |
| Li M /2011  (2010) | Beijing city  (eastern region) | Version of 2004 | middle school students | 1232 | 198 | 16.07 | — | — | — | — | 6 |
| Shi XY/2011  (2010) | Wenzhou city  (eastern region) | Version of 1996 | middle school students | 1425 | 739 | 51.86 | 433/799 | 306/626 | — | — | 6 |
| Shen Y/2011  (-) | Shanghai city  (eastern region) | Version of 2004 | Primary and middle school students | 1437 | 80 | 5.57 | 47/732 | 33/705 | — | — | 5 |
| Chen D/2011  (2010) | Shanghai city  (eastern region) | Version of 1996 | Primary school students | 1500 | 176 | 11.73 | 90/694 | 86/806 | — | — | 6 |
| Shi ZhF/2011  (2008) | Jinan city  (eastern region) | Version of 1996 | Primary school students | 2212 | 332 | 15.01 | 192/1170 | 140/1042 | 332/2212 | — | 5 |
| Jiang W/2011  (2010) | Nanning city  (western region) | Version of 1996 | 2-12 years old | 2584 | 675 | 26.12 | 407/1467 | 268/1117 | — | 675/2584 | 6 |
| Jiang W/2011  (2009) | Nanning city  (western region) | Version of 1996 | 2-6 years old | 2943 | 762 | 25.89 | 440/1639 | 322/1304 | — | — | 6 |
| Zhang JW/2011  (2009) | Shanghai city  (eastern region) | Version of 1996 | Primary and middle school students | 3104 | 1732 | 55.8 | 1004/1648 | 728/1456 | — | — | 6 |
| Wang P/2011  (2007) | Liuzhou city  (western region) | Version of 1996 | Primary and middle school students | 3261 | 368 | 11.28 | 223/1537 | 145/1724 | 268/2171 | 401/1090 | 6 |
| Zhu XX/2011  (2007) | Hangzhou city  (eastern region) | Version of 1996 | Primary and middle school students | 3518 | 1457 | 41.42 | 914/2042 | 543/1476 | — | — | 6 |
| Wei L/2011  (2009) | Ningbo city  (eastern region) | Version of 1996 | Primary and middle school students | 3803 | 767 | 20.17 | 446/2128 | 321/1675 | — | — | 6 |
| Xie XH/2011  (2004) | Beijing city  (eastern region) | Version of 2004 | 0-6 years old | 5528 | 478 | 8.65 | 266/2836 | 2692/212 | 313/2970 | 165/2558 | 7 |
| Hu GQ/2011  (2008) | nationwide | Version of 2004 | 0-4 years old | 10819 | 173 | 1.6 | 106/5902 | 67/4905 | — | — | 6 |
| Liu WL/2012  (2010) | Shandong province  (eastern region) | Version of 1996 | 0-9 years old | 370 | 37 | 10 | — | — | — | 37/370 | 6 |
| Wu MY/2012  (2009) | Shanghai city  (eastern region) | Version of 1996 | 2-6 years old | 598 | 75 | 12.54 | 51/336 | 24/262 | 75(94)/598 | — | 4 |
| Shi RX/2012  (2010) | Beijing city  (eastern region) | Version of 2004 | middle school students | 1862 | 341 | 18.31 | 211/924 | 130/938 | — | — | 6 |
| Wu XH/2012  (2011) | Sheyang county  (eastern region) | Version of 2004 | Primary and middle school students | 1911 | 332 | 17.37 | — | — | — | — | 5 |
| Tang YM/2012  (2010) | Shanghai city  (eastern region) | Version of 2004 | Primary and middle school students | 2610 | 263 | 10.08 | 191/1339 | 72/1271 | — | — | 5 |
| Sun Y/2012  (2009) | Changfeng county  (middle region) | Version of 2004 | Primary and middle school students | 2917 | 426 | 14.6 | 255/1533 | 171/1384 | — | 426/2917 | 8 |
| Yang DY/2012  (2011) | Cangnan county  (eastern region) | Version of 1996 | Primary and middle school students | 4926 | 1278 | 25.94 | 741/2795 | 537/2131 | 611/2529 | 667/2397 | 7 |
| Zhang L/2012  (2006) | Hubei province  (middle region) | Version of 1996 | 0-14 years old | 10688 | 2028 | 18.97 | 1177/5717 | 851/4971 | 770/5894 | 1258/4794 | 6 |
| Hu M/2012  (2008) | nationwide | Version of 2004 | 5-15 years old | 21973 | 374 | 1.7 | 248/11547 | 126/10413 | — | — | 6 |
| Yang J/2013  (2012) | Guangzhou city  (eastern region) | Version of 2004 | middle school students | 437 | 170 | 38.9 | 95/247 | 75/190 | — | — | 4 |
| Sun N/2013  (2012) | Xianyang City  (western region) | Version of 1996 | Kindergarten children | 608 | 221 | 36.35 | 127/318 | 94/290 | 221/608 | — | 6 |
| Wang XX/2013  (2013) | Dalian city  (eastern region) | Version of 1996 | Primary and middle school students | 1023 | 229 | 22.39 | 137/526 | 92/497 | — | — | 5 |
| Li GC/2013  (2011) | Luquan County  (western region) | Version of 1996 | 0-6 years old | 1054 | 312 | 29.6 | 216/594 | 96/460 | — | 312/1054 | 4 |
| Yao YH/2013  (2011) | Shanghai city  (eastern region) | Version of 1996 | 0-14 years old | 1520 | 22 | 1.45 | — | — | 22/1520 | — | 7 |
| Zhang MR/2013  (2011) | Kunming city  (western region) | Version of 1996 | 0-17 years old | 2151 | 113 | 5.25 | 84/1090 | 29/1061 | — | 62/1212 | 7 |
| Zhou Y/2013  (2012) | bengbu city  (middle region) | Version of 1996 | middle school students | 2530 | 1097 | 43.36 | 569/1217 | 528/1313 | 603/1484 | 494/1046 | 7 |
| Hu MJ/2013  (2003) | Gaungdong province  (eastern region) | Version of 1996 | 0-19 years old | 7592 | 2156 | 28.4 | — | — | — | — | 6 |
| He J/2013  (2010) | Qujing City  (western region) | Version of 1996 | Primary and middle school students | 10561 | 2531 | 23.97 | 1490/5456 | 1041/5105 | 2531/10561 | — | 6 |
| Shao QJ/2014  (2007) | Henan province  (middle region) | Version of 1996 | Middle school students and under | 232 | 32 | 13.79 | 23/127 | 9/105 | — | 32/232 | 5 |
| Jia GZh/2014  (-) | Heze City  (eastern region) | Version of 1996 | 3-14 years old | 475 | 190 | 40 | 135/262 | 55/213 | — | 190/475 | 6 |
| Zhao LF/2014  (2013) | Anhui province  (middle region) | Version of 1996 | Primary and middle school students | 1025 | 824 | 80.39 | 383/470 | 441/555 | — | 824/1025 | 5 |
| Yang YL/2014  (2012) | Zibo City  (eastern region) | Version of 1996 | 3-6 years old | 1231 | 81 | 6.58 | 48/632 | 33/599 | — | 81/1231 | 5 |
| Zhang XJ/2014  (2011) | Ningbo city  (eastern region) | Version of 2004 | Primary school students | 1778 | 210 | 11.81 | — | — | 210/1778 | — | 7 |
| Yang YC/2014  (2013) | Zhengzhou city  (middle region) | Version of 2004 | Middle school students | 1987 | 306 | 15.4 | 177/899 | 129/1088 | — | — | 6 |
| Wei YH/2014  (2013) | Guangzhou city  (eastern region) | Version of 1996 | Primary school students | 2430 | 507 | 20.86 | 279/1243 | 228/1187 | — | — | 6 |
| Zhang YX/2014  (2013) | Changzhou city  (eastern region) | Version of 2004 | Middle school students | 2601 | 276 | 10.61 | — | — | — | — | 6 |
| Deng FM/2014  (2013) | Liling City  (middle region) | Version of 1996 | Primary and middle school students | 3257 | 356 | 10.93 | 199/1575 | 157/1682 | — | 356/3257 | 5 |
| Duan JL/2014  (2012) | Beijing city  (eastern region) | Version of 2004 | Primary and middle school students | 16680 | 1650 | 9.89 | — | — | — | — | 6 |
| Nie ShP/2014  (2010) | Guangdong province  (eastern region) | Version of 2004 | Middle school students | 18633 | 2807 | 15.06 | — | — | — | — | 6 |
| Zhang GZh/2015  (2013) | Urumqi Municipality  (western region) | Version of 1996 | 3-6 years old | 258 | 48 | 18.6 | 28/145 | 20/113 | 48/258 | — | 4 |
| Zhang JB/2015  (2011) | Changzhi city  (middle region) | Version of 1996 | 0-14 years old | 510 | 21 | 4.12 | — | — | 21/510 | — | 6 |
| Dou DM/2015  (2012) | Kaifeng City  (middle region) | Version of 1996 | Primary school students | 594 | 260 | 43.77 | — | — | — | 260/594 | 5 |
| Liu HY/2015  (2012) | Guangzhou city  (eastern region) | Version of 1996 | 3-6 years old | 612 | 214 | 34.97 | — | — | — | — | 4 |
| Wang L/2015  (-) | Taiyuan City  (middle region) | Version of 2004 | Primary school students | 618 | 300 | 48.54 | — | — | 300/618 | — | 6 |
| Zhang H/2015  (2013) | Daqing city  (middle region) | Version of 1996 | 1-14 years old | 750 | 125 | 16.67 | 78/412 | 47/338 | 125/750 | — | 5 |
| Zhang ShL/2015  (2015) | Jiamusi City  (middle region) | Version of 1996 | Middle school students | 802 | 407 | 50.75 | 232/423 | 175/379 | 407/802 | — | 5 |
| Qu ShX/2015  (2012) | Shanghai city  (eastern region) | Version of 1996 | 3-6 years old | 936 | 236 | 25.21 | 122/457 | 114/479 | 236/936 | — | 7 |
| Luo J/2015  (2013) | Hanchuan city  (middle region) | Version of 2004 | Primary and middle school students | 1828 | 397 | 21.72 | 279/1112 | 118/716 | — | 397/1828 | 7 |
| Guo L/2015  (2013) | AnYang city  (middle region) | Version of 2004 | Middle school students | 2142 | 245 | 11.44 | — | — | — | — | 7 |
| Tao X/2015  (2013) | Enshi City  (middle region) | Version of 2004 | Primary and middle school students | 2196 | 384 | 17.49 | 233/1128 | 151/1068 | — | 384/2196 | 6 |
| Ya F/2015  (2010) | Xiamen city  (eastern region) | Version of 1996 | Primary and middle school students | 2816 | 303 | 10.76 | 178/1382 | 125/1434 | — | — | 6 |
| Li XM/2015  (2015) | Yunnan province  (western region) | Version of 1996 | Primary and middle school students | 2909 | 909 | 31.25 | 468/1415 | 441/1494 | 110/607 | 799/2302 | 7 |
| Zhang HZh/2015  (2011) | Shenyang city  (eastern region) | Version of 1996 | Middle school students | 3477 | 1051 | 30.23 | 548/1599 | 503/1878 | — | — | 7 |
| Sun QL/2015  (2013) | Shenzhen city  (eastern region) | Version of 2004 | Primary school students | 5834 | 1458 | 24.99 | — | — | 1458/5834 | — | 5 |
| Peng ZhH/2015  (2010) | Shenzhen city  (eastern region) | Version of 1996 | 2-6 years old | 6175 | 798 | 12.92 | 489/3362 | 309/2813 | 798/6175 | — | 5 |
| Liu XX/2016  (2015) | Shanghai city  (eastern region) | Version of 1996 | Primary school students | 1254 | 466 | 37.16 | — | — | — | — | 6 |
| Guo Q/2016  (-) | Enshi city  (eastern region) | Version of 2004 | Middle school students | 1917 | 297 | 15.5 | — | — | — | — | 7 |
| Yang XW/2016  (2013) | Hunan province  (middle region) | Version of 1996 | School-age children | 1083 | 661 | 61 | 365/546 | 296/537 | — | 661/1083 | 5 |
| Liu X/2016  (2013) | Jining city  (eastern region) | Version of 2004 | Middle school students | 686 | 197 | 28.71 | — | — | — | — | 5 |
| Chang YH/2016  (2015) | Luzhou city  (western region) | Version of 2004 | Middle school students | 16465 | 2180 | 13.24 | 1259/8573 | 921/7892 | 586/4803 | 1594/11662 | 7 |
| Sun GX/2016  (2015) | Xuzhou city  (eastern region) | Version of 1996 | Middle school students | 572 | 190 | 33.22 | 115/294 | 75/278 | — | 190/572 | 6 |
| Hao ZhH/2016  (-) | Taiyuan city  (middle region) | Version of 2004 | 8-13 years old | 2854 | 660 | 23.1 | 410/1487 | 250/1367 | — | — | 7 |
| Sheng JH/2016  (-) | Luliang county  (western region) | Version of 2004 | 10-14 years old | 1631 | 335 | 20.5 | 205/865 | 130/766 | 109/418 | 226/1213 | 8 |
| Haung Y/2016  (-) | Yunnan province  (western region) | Version of 1996 | Primary and middle school students | 2909 | 909 | 31.7 | — | — | — | 909/2909 | 4 |
| Lian XX/2017  (-) | Aba Autonomous Prefecture  (western region) | Version of 2004 | 3-6 years old | 4368 | 657 | 15.04 | 361/2213 | 296/2155 | — | — | 4 |
| Ren YP/2017  (-) | Shanghai city  (eastern region) | Version of 2004 | 6-12 years old | 6093 | 1401 | 22.99 | 776/3121 | 625/2972 | — | — | 6 |
| Zhu J/2017  (-) | Shaoguan city  (eastern region) | Version of 1996 | 0-6 years old | 3000 | 400 | 6.67 | 238/1629 | 162/1371 | — | — | 4 |
| Zhang XZh/2017  (2015) | Wenzhou city  (eastern region) | Version of 2004 | Middle school students | 5199 | 920 | 17.69 | 510/2726 | 410/2473 | — | — | 6 |
